# Isolated neonatal rat papillary muscles: a new model to translate neonatal rat myocyte signaling into contractile mechanics

**DOI:** 10.14814/phy2.12694

**Published:** 2016-02-11

**Authors:** Damir Nizamutdinov, Hao Feng, Fnu Gerilechaogetu, Joseph A. Dostal, Donald M. Foster, Shannon S. Glaser, David E. Dostal

**Affiliations:** ^1^ Department of Medical PhysiologyCollege of MedicineTexas A&M University System Health Science CenterTempleTexas; ^2^ Department of OphthalmologyUT Health Science Center San AntonioSan AntonioTexas; ^3^ Central Texas Veterans Health Care SystemTempleTexas; ^4^ Division of GastroenterologyDepartment of Internal MedicineBaylor Scott & White Health Care SystemTempleTexas

**Keywords:** Cardiac contractility, isolated papillary muscle, neonatal cardiac tissue

## Abstract

Isolated cardiac tissue allows investigators to study mechanisms underlying normal and pathological conditions, which would otherwise be difficult or impossible to perform in vivo. Cultured neonatal rat ventricular cardiac myocytes (NRVM) are widely used to study signaling and growth mechanisms in the heart, primarily due to the versatility, economy, and convenience of this in vitro model. However, the lack of a well‐defined longitudinal cellular axis greatly hampers the ability to measure contractile function in these cells, and therefore to associate signaling with mechanical function. In these methods, we demonstrate that this limitation can be overcome by using papillary muscles isolated from neonatal rat hearts. In the methods we describe procedures for isolation of right ventricular papillary muscles from 3‐day‐old neonatal rats and effects of mechanical and humoral stimuli on contraction and relaxation properties of these tissues.

## Introduction

The neonatal rat cardiac ventricular myocytes culture (NRVM) is a popular in vitro model (Golden et al. 2012) which has been used to study a wide variety of processes such as apoptosis, oxidative stress, hypertrophic growth, signal transduction, and transcriptional regulation (Gerilechaogetu et al. 2013; Jenie et al. 2013; Lal et al. 2013; Lucchese et al. 2013; Yu et al. 2014; Zhou and Lu 2013). The utility of these cells has been recently extended by combining isolated NRVM and nonmyocytes with various matrix proteins to form three‐dimensional (3‐D) scaffolds, which after several days in culture form functional cardiac tissue that can be used for cardiac repair and contractile function studies (Ikonen et al. 2013; Kawaguchi et al. 2013). The use of 3‐D scaffolds provide a convenient means to study contractile responses, however the cardiac tissue phenotype is altered by the type of matrices, combination of cell types and culture conditions (Li et al. 2012; Ikonen et al. 2013). As an alternative, we propose the use of 0‐to 3‐day‐old neonatal rat papillary muscles, which contain all the characteristics of a physiologically relevant 3‐D muscle microenvironment. Papillary muscles therefore accurately reflect the mechanical and elastic characteristics of in vivo heart tissue, as well as functional electrophysiological unity of the cardiac cells. Adult mouse and rat papillary muscles preparations have been used to complement isolated adult myocyte preparations. However, to our knowledge, no such model has been developed for neonate heart tissue. In the following methods, we describe the isolation procedures and neonatal rat papillary muscle responses to electrical, mechanical, and beta‐adrenergic stimuli.

## Materials and Methods

### Hardware, surgical instruments, and reagents

Major equipment used in these studies included a small muscle contractile apparatus (Aurora Scientific, Inc., Aurora, ON, Canada), equipped with an Aurora Scientific Force Transducer (405A), field stimulator‐(IonOptix MyoPacer Field Stimulator [MYP 100]), and a temperature controller‐(IonOptix Thermometer/TEC Controller) connected through an analog‐to‐digital hardware interface system (Fluorescent System Interface, IonOptix) and monitored in real‐time using computer software (IonWizard 6.2, IonOptix, Milton, MA). Although the Aurora Scientific Small Intact Muscle Test System was used to measure contractile responses of papillary muscles, the methods described below apply to other small muscle test systems. Dissections and papillary muscle manipulations were carried out using a stereo boom microscope (AmScope, 3.5× – 90× magnification, model SM‐4BZ‐FRL) equipped with a direction‐variable 80 led ring illuminator (AmScope, LED‐80AM, Irvine, CA). Attachment of papillary muscles to the small muscle test system and monitoring of contractile responses were visualized using a wide‐field, wide‐base stereo‐zoom microscope (AmScope, 3.5× – 90× magnification, model SM‐1TZ‐FOD), equipped with a 150 watt fiber optic dual gooseneck illuminator with dimmer (AmScope, HL250‐AOYS) with additional bottom lighting (Artograph, LED LightPad A290, Delano, MN) and 10 mega pixel digital camera (ToupTek, UCMOSO09000KPA). Surgical instruments used were as follows: Vannas spring curved surgical scissors (2 mm cutting edge), Dumont #5 SF surgical forceps (0.025 × 0.005 mm), Moria fine scissors, standard pattern forceps with serrated tips (Fine Science Tools), and micro surgical braided silk suture (size 8–0). Reagents and solutions used: ampicillin (100×) solution, low‐glucose Dulbecco's Modified Eagle's medium (DMEM), fetal bovine serum, horse serum, 10 × medium 199 (Gibco, Life Technologies, Carlsbad, CA), and sodium bicarbonate 99.5% (Fisher Scientific‐MP Biomedicals, Pittsburgh, PA).

### Isolation of neonatal rat papillary muscles

Papillary muscles were isolated from the right ventricles of 3‐day‐old neonatal Sprague–Dawley rat pups. Procedures on research animals were conducted with approval of the Baylor Scott & White Animal Care and Use Committee in accordance with the recommendations of the Guide for the Care and Use of Laboratory Animals (Eighth edition) revised by the National Research Council in 2011 (published by the National Academies Press), National Institutes of Health and American Association for the Accreditation of Laboratory Animal Care (AAALAC) guidelines and the Public Health Service (PHS) Policy on Humane Care and Use of Laboratory Animals.

**Figure 1:**
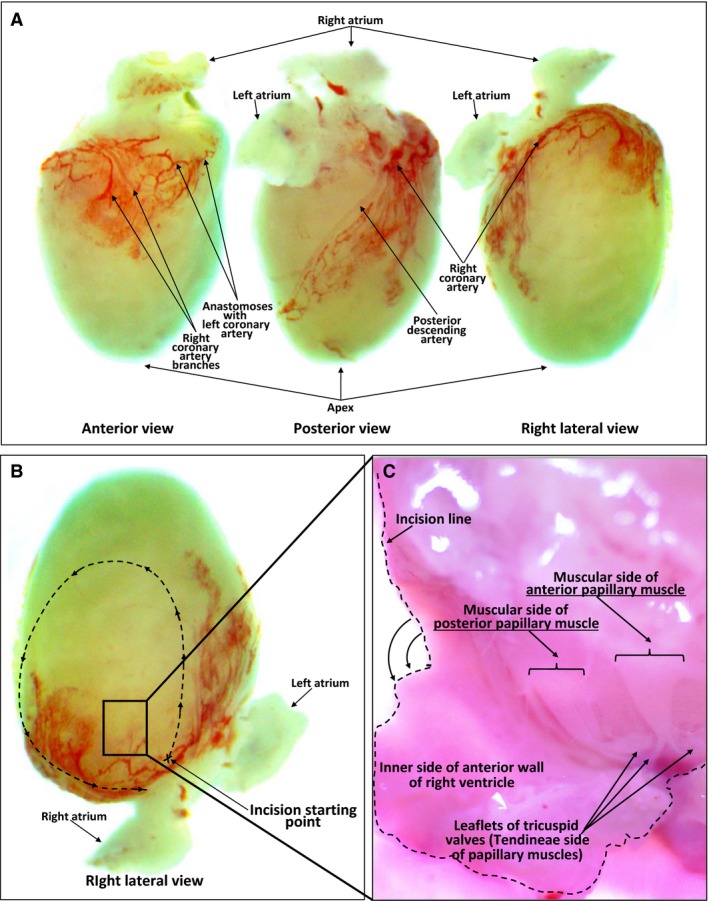
Anatomy and isolation of right ventricular papillary muscles in the neonatal rat heart. (A) Anterior, posterior and lateral views of a 3‐day‐old neonatal rat heart. (B) Orientation of the heart prior to exposure of the right ventricular chamber. The starting point and direction of incision are indicated by the dashed line. (C) Interior of the right ventricle. The septal surface showing posterior and anterior papillary muscle attachment sites.

Following euthanasia (Golden et al. 2012), hearts were removed and placed into a 100 × 20 mm sterile culture dish with chilled (4°C) isolation media (DMEM and M 199 medium in a 4:1 ratio, supplemented with 10% horse serum, 5% fetal bovine serum, and 34 *μ*g/mL ampicillin, pH to 7.4, and filter sterilized). The isolated heart was positioned for easy access to the right ventricle (Fig. 1A, B), after which a stereo microscope was used to expose the right ventricular chamber, and remove the papillary muscle. To gain access to inside of the right ventricular chamber, an incision was started at the base of the ventricles (Fig. 1B) using a small scissor and was extended toward the apex of the heart (Fig. 1B). After completion of the 360° incision, the anterior wall of right ventricle was removed, thus exposing the three right ventricular papillary muscles (Fig. 1C). Care was taken not to physically damage papillary muscles by stretching or crushing the tissue during the isolation procedure. We found that less trauma occurred when the anterior and posterior papillary muscles were first detached from the tricuspid valve insertion sites (chordae tendineae end) (Fig. 1C) followed by removal of the wide muscular end of the papillary muscle from the ventricular wall. High‐quality stainless‐steel scissors and forceps were used for these procedures. Stepwise details of these isolation procedures are available in the Supplemental_Detailed Methods in Data S1.

### Preparation of the small intact muscle test system

**Figure 2:**
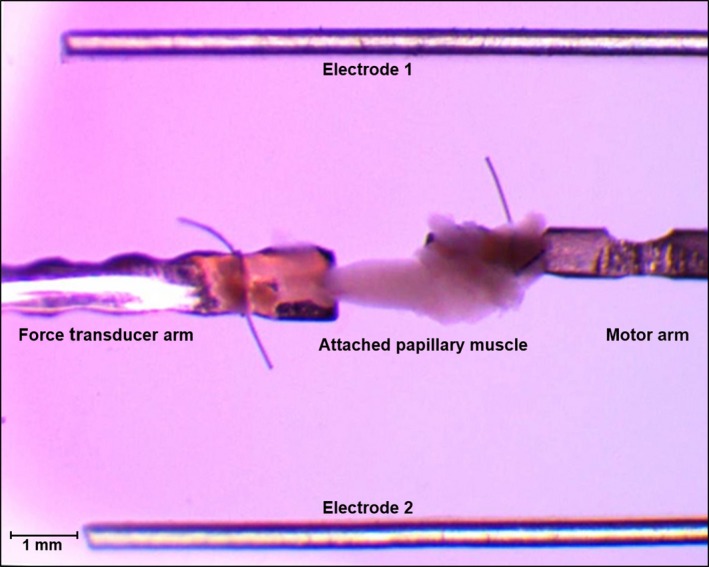
Pacing chamber of the small intact muscle test system with attached papillary muscle. The papillary muscle was attached to the test system using premade double‐overhand knot suture loops. The chordae tendineae (valvular end) was attached to the force transducer adapter arm (left) and the opposite end of the muscle (muscular end) was attached to the motor adapter arm (right). The muscle test chamber was filled with isolation medium (600 *μ*L) prior to attachment of the papillary muscle to adapter arms of the apparatus.

Prior to contractile studies, the pacing chamber of the small intact muscle test system was prepared for attachment of the papillary muscle to the force transducer by washing 2–3 times and being filled with 600 *μ*L of isolation media. Experiments performed in the 600 *μ*L bath of media, which was partially (200 *μ*L) replaced every 5 min to ensure constant pH in the media and oxygenation of the papillary muscle. When effects of pharmacological agents were evaluated, 200 *μ*L of 1× freshly prepared drug solution in fresh media was replaced every 5 min in order to maintain constant drug concentrations under physiologic conditions. Mounting of the muscle onto the test system was facilitated by previous insertion of simple double‐overhand suture loops (micro surgical braided silk suture [size 8–0]) over the motor adapter and force transducer arms (Fig. 2). Before muscle attachment, the two mounting arms were aligned to the same horizontal (*X*–*Y* planes) and vertical axes (*Z*‐plane) using corresponding micrometers, in which a small gap (5–10 *μ*m) was left between the opposing arm tips.

### Attachment of papillary muscle to the force transducer and motor adapter arms

Following preparation of the experimental chamber, isolated papillary muscle were transferred to the muscle contraction test chamber. Using premade suture loops the chordae tendineae end and the wide muscular end of the papillary muscle were attached to the force transducer and motor adapter arms, respectively (Fig. 2). Once attached, the papillary muscle was aligned in the *y*‐ and *z*‐axis and optimally stretched (preloaded) in the horizontal axis (*x*‐axis) using the motor‐arm micrometer. Preload was adjusted to the length of the muscle bundle (contractile portion) prior to removal from the heart. Contractile force measured at that muscle length was considered optimal and served as a starting point for the stretch‐related experiments.

### Preparation of attached papillary muscle for functional studies

Pacing parameters and tension (muscle preload) were adjusted prior to experimentation, starting with initial pacing at 0.5 Hz (30 beats/min), and using synchronous electrical field stimulation. The electrical stimulator was initially set to a direct‐current bipolar pulse‐wave of 0 V with a 5 msec duration. The voltage was increased by 0.5 V increments every 10–20 sec until papillary muscles responded to each stimulus. While pacing, muscle tension (preload) was increased until consistent contractile responses (with regard to pattern and peak height) were obtained. Baseline of the contractile signal was electronically adjusted through the force transducer offset controller. The muscles were paced for ~30 min at 1 Hz, prior to experimentation. A movie depicting contraction of a paced papillary muscle at this stage of the setup is shown in the Supplemental_Video in Video S1.

### Measurements of force–length relationship using papillary muscle

**Figure 3:**
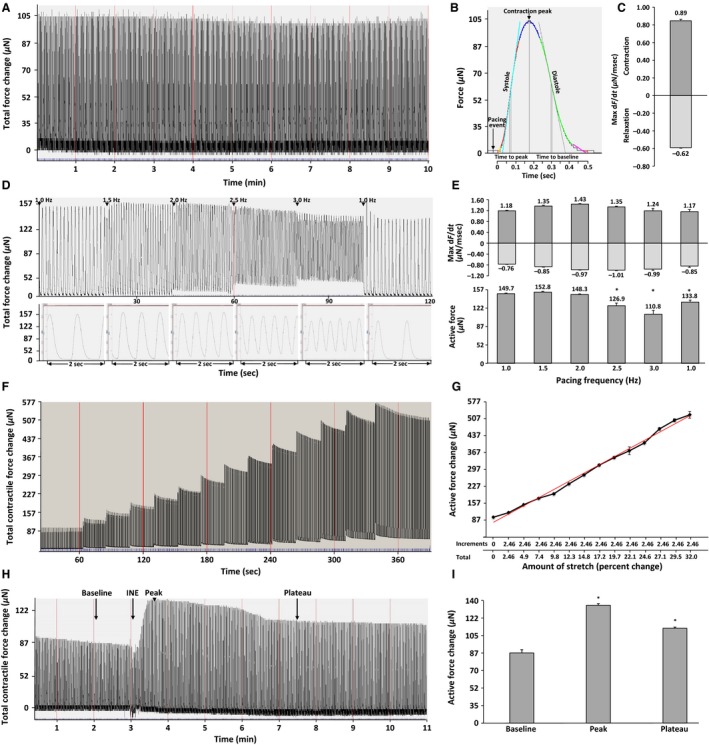
Effects of mechanical stretch, pacing, and adrenergic stimulation on the contractile performance of isolated neonatal rat papillary muscles. All experiments were performed at room temperature (24°C). The papillary muscles were bathed in isolation media and contractile responses were recorded and analyzed using IonOptix Wizard 6.2 software. Gray vertical lines in the chart tracing correspond to 1 min intervals. (A) Representative recording of a papillary muscle paced for 10 min at 1.0 Hz (60 beats/min) using a bipolar wave form set at 2.0 V with a duration of 5 milliseconds (msec). (B) Morphology of a single contractile cycle. The pacing event mark, maximal peak contractile response, time to peak (systole), and time to baseline (diastole) in msec. Force measurements are expressed in micronewtons (*μ*N). (C) Papillary muscle contractile and relaxation velocities. Maximum force velocity during contraction (Max +d*F*/d*t*) and relaxation (Max −d*F*/d*t*) phases in normal contractile cycle of neonatal rat papillary muscle paced at 1 Hz. Means ± SEMs are representative of 20 contractile cycles. Force–velocity units are expressed in *μ*N/msec. (D) Frequency‐dependent contractile responses. The dependence of pacing frequency on force generation was determined by increasing the pacing frequency from 1.0 Hz (60 bpm) to 3.0 Hz (180 bpm) in 0.5 Hz (30 bpm) increments, after which the pacing frequency was returned to 1.0 Hz. The muscle was paced for 20 sec at each frequency increment. Bottom panel: The morphology of the contractile force peaks obtained in each pacing frequency is shown for a duration of 2 sec. Details can be found in the “Supplemental_Tables” file (Table S1). This experiment was repeated four times using papillary muscles isolated from four different animals. (E) Maximum values of force rate change (top) and contractile force generated by muscle (bottom) during contraction/relaxation cycle at corresponding pacing frequency. The bar graphs show Max +d*F*/d*t* and Max −d*F*/d*t* during contraction and relaxation. The force velocity units are expressed as *μ*N/msec. (top panel). The bar graphs (bottom panel) show changes in active contractile forces generated at the different pacing frequencies. The means ± SEMs represent the first 10 contractile cycles after each increment in the pacing frequency. Details can be found in the “Supplemental_Tables” file (Table S1). *P < 0.001, in which frequency increment values for departure/return velocities and active contractile force measurements were compared to those obtained at 1.0 Hz. (F) The effects of mechanical stretch on papillary muscle contraction were determined using a pacing frequency of 1.0 Hz. Increases in stretch were applied using a precision micrometer and contractile responses were recorded for ~20 sec per step. The depicted papillary muscle was stretched from an initial length of 2.0 mm to a final length of 2.64 mm (32%), over 13 stretch steps (with 2.46% of stretch increment per step). This experiment was repeated four times using papillary muscles isolated from four different animals. (G) The line graph, with regression analysis shows the relationship between average values of percent stretch and active force (depicted in *μ*N) generated by the papillary muscle. The means ± SEMs represent the first 10 contractile cycles after each stretch increment. (H) Effects of β_1_‐adrenergic stimulation on contractile responses. Temporal effects of 10 μmol/L isoproterenol (INE) on papillary muscle contractile performance. Contractile responses were recorded at 1.0 Hz (60 bpm) over 8 min. In addition to time of INE administration, the arrows indicate the baseline, peak contractile response to INE, and subsequent plateau response. Details can be found in the “Supplemental_Tables” file (Table S2). This experiment was repeated four times on papillary muscles isolated from four different animals. (I) Active forces generated before and after INE administration. The bar graph depicts means ± SEMs of the first 10 contractile cycles after the indicated time points. Details can be found in the “Supplemental_Tables” file (Table S2). *P < 0.001 between the baseline and peak or plateau responses. This experiment was repeated four times using papillary muscles isolated from four different animals.

Isometric force–length relationships are conveniently used to characterize systolic and diastolic contractile properties of cardiac muscle. These relationships are measured while holding the ends of an isolated muscle and measuring the force developed at different muscle lengths while preventing the muscle from shortening. The muscle preparation used in these methods described above is therefore an isometric force generation model, since the papillary muscles were directly attached to the force transducer and immovable motor arm. To determine the effects of tension (preload) on isometric force generation, the muscle was successively stretched by 5.0 micron increments using the motor arm micrometer. At baseline and following each stretch, length of the muscle was microscopically determined by measuring only the undamaged contractile portion of the tissue (Fig. 2), in which a typical end‐systolic force–length relationship is shown in Figure 3F. Interestingly, the neonatal rat papillary muscles did not give true isometric responses, as contraction of the papillary muscle stretched the chordae tendineae, resulting in some muscle shortening. Although a limitation of this model, measurements of simultaneous changes in muscle length and force during contraction can be to determine cardiac work, which is calculated as force × distance.

### Statistics

Data are presented as means ± the standard error of the mean (SEM). Significant differences among groups were estimated by one‐way ANOVA followed by Tukey's multiple comparison test (Instat 3.06, GraphPad Software Inc., La Jolla, CA). Experiments were repeated four times from four papillary preparations from four animals and a value of *P* < 0.05 was considered statistically significant.

## Results

### Effects of mechanical, electrical and humoral stimulation on contractile performance of isolated neonatal rat papillary muscles

#### Electrical pacing of isolated neonatal rat papillary muscles

Figure 3A shows typical contractile responses of isolated papillary muscle recorded 30 min after mounting in the test apparatus and paced at 1 Hz. The papillary muscle responded to all electrical stimuli (1 Hz, 2 V) within the 10 min test period, and generated consistent contractile responses with similar magnitudes (base to peak) at room temperature (24°C) (Fig. 3B). Maximum contractile responses and relaxation velocities of paced papillary muscles were also recorded (Fig. 3C).

#### Frequency responses of isolated neonatal rat papillary muscle

The cardiac muscle can dynamically change its rate of contraction (Blinks and Koch‐Weser 1961; Edman and Johannsson 1976; Lewartowski and Pytkowski 1987; Endoh 2004; Janssen 2010). The force–frequency relationship (FFR) is an important intrinsic regulatory mechanism of cardiac contractility (Janssen 2010). The increase in contractile force associated with increasing frequency is thought to reflect cardiac contractile reserve and is reported to be different depending on the range of frequency, age, and species of the animal (Endoh 2004). The frequency‐dependent changes in systolic and diastolic force, along with intracellular calcium transients have been used to assess the effects of pharmacologic agents and disease states on cardiac excitation‐contraction coupling (Schwinger et al. 1993). We therefore examined the effects of pacing frequency on contractile force in neonatal rat papillary muscles. After 30 min of baseline pacing at 1.0 Hz the pacing rate was increased to 3.0 Hz (180 bpm) in 0.5 Hz increments per frequency step (in 4 steps total). The papillary muscle was stimulated for 20 sec at each frequency. The pacing rate was then adjusted to the initial frequency of 1.0 Hz (Fig. 3D). Increasing the pacing frequency from 1.0 to 1.5 Hz resulted in a positive FFR with increased contractile force, which then declined at higher pacing frequencies (Fig. 3D, E). The contraction–relaxation velocity of muscle increased until the pacing frequency reached 2.0 Hz, after which it began declining at higher frequencies (Fig. 3E). Therefore, the frequency associated with an initial increase in contractile force with followed by a secondary‐phase of negative FFR, consistent with previous studies using adult rat heart tissue (Endoh 2004). The secondary‐phase negative FFR in the rat heart has been postulated to be due to complex cellular mechanisms of decreased myofilament calcium ion (Ca^2+^) sensitivity due to a decrease in intracellular pH (Morii et al. 1996), the refractoriness of sarcoplasmic reticulum (SR) Ca^2+^ release channels (Meyer et al. 1999; Maier and Bers 2002), and increased SR Ca^2+^ ATPase (SERCA2a) activity due to excision and preparation of cardiac tissue for in vitro experiments (Taylor et al. 2004).

#### Stretch responses of isolated neonatal rat papillary muscle

Cardiac muscle responds to increased preload (Allen and Kentish 1985) (mechanical stretch) by increasing force and velocity of contraction; known to be the Frank–Starling mechanism (Patterson et al. 1914; Chantler et al. 2012; Dori et al. 2012; Ribaric and Kordas 2012). Altering the preload can be used to assess functional performance, as well as study mechanosensing and excitation‐contractile mechanisms in papillary muscles (Patterson et al. 1914). The neonatal rat papillary muscles were found to demonstrate excellent contractile responses which increased with increasing preload. Figure 3E demonstrates a typical Frank–Starling response in which a papillary muscle was successively stretched in 5.0 micron increments. For each “stretch step”, the contractile responses were recorded for 20 sec (Fig. 3F, G). Following 13 stretch increments, the papillary muscle was stretched 32%, in which the muscle generated greater than a 750% increase in contractile force.

#### Effects of Isoproterenol on isolated neonatal rat papillary muscle performance

The effects of pharmacological agents on the papillary muscle can be evaluated by using agents typically used for studies in plated cells (Bristow et al. 1982). Time‐course effects of a nonselective beta‐adrenergic agonist (10 μmol/L isoproterenol) on neonatal rat papillary muscle contractile performance is shown in Figure 3H. In this example, contractile responses generated by the papillary muscle paced at 1.0 Hz (60 bpm) are shown before and after isoproterenol treatment. As a result, contractility of papillary muscle (Baseline) almost doubled within the first 25 sec of isoproterenol treatment (Peak) and continued at high performance for the next 2.5 min with subsequent stabilization (Plateau) of contractile performance at 37% higher compared to initial contractility (Fig. 3I).

## Discussion

Neonatal rat cardiac cells have proven to be excellent tools for studying basic cardiac physiology and testing of therapeutic agents. When it comes to the regulatory mechanisms of molecular signaling in the heart, studying intracellular ion exchange, and testing different receptors and pharmacological tools, the vast majority of current research is performed with cells isolated from neonatal rat hearts (cardiac myocytes and cardiac fibroblasts) (Gerilechaogetu et al. 2013; Jenie et al. 2013; Lal et al. 2013; Lucchese et al. 2013; Yu et al. 2014; Zhou and Lu 2013). Our results with neonatal papillary muscles indicate that neonatal rat heart tissue is not limited to primary cell cultures, but can be easily extended to the study of contractile function. The isolated heart muscle preparation has several advantages over single‐cell experiments in the study of contractile function of the heart (Spurgeon et al. 1990; Backx and Ter Keurs 1993; Janssen and Hunter 1995; Bers 1997; Janssen et al. 1998). Therefore, it is important to develop a contractile functional model that is efficient, reproducible, resilient, not technically challenging, and can display mechanisms of cellular regulation similar to that observed in isolated cardiac cells. Development of the neonatal rat papillary muscle model is an important advancement since it will allow investigators to couple signaling events to contractile performance in NRVM.

Due to fact that an increase in force development is correlated with an increase in twitch duration (Janssen and de Tombe 1997), interventions that impair relaxation of cardiac muscle such as acute ischemia (Palacios et al. 1978) and congestive heart failure (Grossman 1991) might be also investigated using this model. For the functional comparison to the existing adult murine papillary muscle model all tests were performed at room temperature.

In previous studies, Krebs–Henseleit buffer has been commonly used during experiments (Mulieri et al. 1989; Janssen et al. 1998). In these methods, cell culture media (isolation media) was used as a buffer system and balanced energy source (contains all the necessary amino acids, nutrients, and stable pH), thereby providing an excellent environment for the contracting papillary muscle. Media in the bath was partially replaced every 5 min. This was done to achieve a constant pH in the bath and diminish oxidative stress of the isolated muscle. Taken together, isolation media can serve as a replacement for the existing Krebs–Henseleit buffer system, since it mimics in vitro cell culture conditions used for NRVM.

The contractile state of the cardiac muscle is regulated by a change in the intracellular Ca^2+^ concentration ([Ca^2+^]_i_), regardless of the development stage (Bers 2002; Colella et al. 2008). Likewise, it has been well established that cardiac contractile performance is highly dependent on sarcomeric length (SL), thus a subtle change of ∼100 nm causes a substantial change in myocardial contractility (Allen and Kentish 1985; Fukuda et al. 2001; Hanft et al. 2008). This intrinsic nature of cardiac myofilaments requires an accurate measurement of SL to enhance our understanding of myocardial dynamic properties, not only in adults but also in neonates whose cardiac myocytes undergo rapid growth (Jacot et al. 2009, 2010). However, because of sarcomere dynamics and the lack of technology to measure single SL with high spatial and temporal resolution, the impact on myocyte motion remains to be systematically investigated, especially in neonatal cardiac myocytes (Shintani et al. 2014).

The passive force–length relationship in skeletal muscle has been investigated for decades, and it shows increasing levels of force at increased muscle lengths. It has been proposed that most of the passive force in skeletal muscle is produced by the giant protein titin, which spans the half‐sarcomere, and connects the A‐band to the I‐bands and Z‐lines (Kellermayer et al. 1997; Rief et al. 1997). Recent evidence shows that the PEVK segments of titin bind Ca^2+^ with high affinity (Tatsumi et al. 2001), and that increasing Ca^2+^ concentration enhances force produced by titin at a given sarcomere length (Labeit et al. 2003). This suggests a link between Ca^2+^‐induced activation and force produced by titin (Fig. 3F).

The rapid development and immaturity of neonatal cardiac myocytes, specifically sarcoplasmic reticulum (SR) which grows in density, can explain results observed in Figure 3D and E where the FFR switch occurs from positive to negative at ~1.5 Hz. This observation is different from one reported in thin adult cardiac trabeculae where a FFR switch from positive to negative occurs at ~10 Hz. This could be partly due to the diameter (width) of the muscles, and is one of the limitations of the response to increased stimulus rate.

In summary, the above observations indicate that isolated neonatal rat papillary muscle responds very well to electrical, mechanical and β‐adrenergic stimulations and therefore forms an excellent model to study cardiac function and to translate signaling responses to the study of contractile function in the neonatal rat heart. Isolated adult mouse and rat papillary muscles preparations have been established to complement isolated adult myocyte preparations. However, to our knowledge, this is the first papillary model developed for the neonate rat heart. We therefore feel that the neonatal rat papillary muscle model described in this study will be beneficial to laboratories that routinely use neonatal rat myocytes and therefore represents a significant contribution to the cardiac field.

## Conflict of Interest

None declared.

## Supporting information




**Table S1.** Departure, return velocity and contractile force responses based on changes in the pacing frequency. **Table S2.** Changes in contractile force in response to 10 µmol/L isoproterenol.

**Video S1.** Contractility of paced isolated neonatal rat papillary muscle.

**Data S1.** Detailed methods description.
