# Supplementary material for: Isolated neonatal rat papillary muscles: a new model to translate neonatal rat myocyte signaling into contractile mechanics
Source: Physiol Rep. 2016 Feb 11;4(3):e12694. doi: 10.14814/phy2.12694 (PMC4758931; doi:10.14814/phy2.12694)
Supplement: Supplementary file 1 — Table S1. Departure, return velocity and contractile force responses based on changes in the pacing frequency. Table S2. Changes in contractile force in response to 10 µmol/L isoproterenol. [file PHY2-4-e12694-s001.docx]

**Tables**

**Table 1.** Departure, return velocity and contractile force responses based on changes in the pacing frequency.

| Frequency (Hz) | Departure velocity (mean ± SEM) (µN/msec) | P-value | Return velocity (mean ± SEM) (µN/msec) | P-value | Contractile force (mean ± SEM) (µN) | P-value |
| --- | --- | --- | --- | --- | --- | --- |
| 1.0 | 1.18 ± 0.006 | - | -0.76 ± 0.003 | - | 149.7 ± 0.443 | - |
| 1.5 | 1.35 ± 0.008 | <0.001 | -0.85 ± 0.006 | <0.001 | 152.8 ± 0.505 | 0.239 |
| 2.0 | 1.43 ± 0.006 | <0.001 | -0.97 ± 0.004 | <0.001 | 148.3 ± 0.365 | 0.899 |
| 2.5 | 1.35 ± 0.009 | <0.001 | -1.01 ± 0.005 | <0.001 | 126.9 ± 1.744 | <0.001 |
| 3.0 | 1.24 ± 0.027 | <0.001 | -0.99 ± 0.014 | 0.999 | 110.8 ± 2.590 | <0.001 |
| 1.0 | 1.17 ± 0.025 | <0.001 | -0.85 ± 0.014 | 0.572 | 133.8 ± 1.343 | <0.001 |

**Table 2.** Changes in contractile force in response to 10 µM isoproterenol.

| Response times | Contractile force (mean ± SEM) (µN) | P-value |
| --- | --- | --- |
| Baseline | 87.5 ± 1.434 | <0.001 |
| Peak | 135.1 ± 0.657 | <0.001 |
| Plateau | 112.1 ± 0.468 | <0.001 |
