# Supplementary material for: Isolated neonatal rat papillary muscles: a new model to translate neonatal rat myocyte signaling into contractile mechanics
Source: Physiol Rep. 2016 Feb 11;4(3):e12694. doi: 10.14814/phy2.12694 (PMC4758931; doi:10.14814/phy2.12694)
Supplement: Supplementary file 3 — Data S1. Detailed methods description. [file PHY2-4-e12694-s003.docx]

**Materials**

***Equipment:***

1. Horizontal Laminar Flow Hood.

2. Dissecting Microscope with 3.5x - 90x magnification (SM-1TRZ) and 10 MP digital camera (UCMO SO 09000KPA).

3. Dissecting Microscope (SM-1TRZ) with light source for papillary muscle fixation.

4. Portable Dual Gooseneck Light System (AmScope).

5. LED Dissecting Light System (AmScope LED 80AM).

6. Portable Bottom Panel Light System (LightPad A930).

7. Aurora Scientific Small Intact Muscle Test System (Aurora Scientific 1500A).

8. Ion Optix MyoPacer Field Stimulator (MYP 100).

9. Tektronix 2 Channels Digital Oscilloscope.

10. Micro Temperature Controller (Ion Optix Termometer/TEC Controler).

11. Computer Module (Dell Optiplex 990).

12. Software for Data Recording and Analysis (Ion Wizard 6.0).

***Surgical instruments:***

1. Vannas Spring Curved Surgical Scissors- 2 mm cutting edge (Fine Science Tools).

2. Dumont #5 SF Surgical Forceps- 0.025 x 0.005 mm (Fine Science Tools).

3. Dumont #5 SF Surgical Forceps- 0.025 x 0.005 mm (Roboz).

4. Moria Fine Scissors (Fine Science Tools).

5. Standard Pattern Forceps- with serrated tips (Fine Science Tools).

6. Hartman Hemostats- with curved, serrated tips (Fine Science Tools).

7. Micro Surgical Suture (size 8-0).

***Isolation procedure reagents, solutions and media:***

1. Ampicillin (100x) solution (Gibco).

2. Low Glucose Dulbecco’s Modified Eagle’s Medium (DMEM, Gibco, Life Technologies).

3. Fetal Bovine Serum (Gibco, Life Technologies).

4. Horse Serum (Gibco, Life Technologies).

5. Medium 199 (10x) (Gibco, Life Technologies).

6. Sodium Bicarbonate 99.5% (Fisher Scientific-MP Biomedicals).

7. Isolation Media: Combine DMEM and M 199 in a 4:1 ratio and supplement with 10% horse serum, 5% fetal bovine serum, and 34 μg/mL ampicillin. pH to 7.4 and filter sterilize.

***Isolation procedure plasticware:***

1. 50-mL sterile conical centrifuge tubes (BD Falcon).

2. 150 x 25-mm sterile culture dishes (Corning).

3. 100 x 20-mm sterile culture dishes (Corning).

4. 60 x 20-mm sterile culture dishes (Corning).

5. 25 mL serological pipets.

6. 500 mL (0.20 μm pore size) bottle top filters (Santa Cruz).

7. 250 mL beaker.

***Animals:***

Care and use of animals were carried out in accordance with National Institutes of Health and American Association for the Accreditation of Laboratory Animal Care (AAALAC) guidelines, and approved by the Scott and White Memorial/Texas A&M University System Health Science Center Institutional Animal Care and Use Committee (IACUC). A Sprague-Dawley rat breeding colony was maintained at the Scott and White animal facility. Neonatal rats (0-3 day old pups) were used for isolation of papillary muscles.

**Methods**

***Preparation for Isolation Procedure:***

1. Chill isolation media at 4 °C prior procedure.

2. Place a 150-mm sterile culture dish in the laminar flow hood, to be utilized during neonatal rat pup euthanasia.

3. Place sterilized surgical instruments in the hood.

4. Prepare liquid nitrogen, as a part of euthanasia process.

5. Place chilled (4 ^o^C) isolation media into 100 x 20 mm (~30 mL) and 60 x 20 mm sterile culture dishes (~10 mL).

***Harvesting Neonatal Rat Hearts:***

1. Following euthanasia, open the chest of the animal with fine scissors and use standard forceps to remove the heart.

2. Transfer the heart to a 60 x 20 mm sterile cell culture dish containing 10 mL of chilled isolation media, which serves to wash out blood from the heart.

3. Use surgical forceps to transfer the heart to a 100 x 20 mm cell culture dish containing 30 mL of 4 °C isolation media and cover with a lid.

4. Transfer the cell culture dish containing the heart under the dissection microscope. With the microscope adjusted to 4.0x magnification (80x total magnification), locate and bring the heart into focal plane.

5. Hold the heart using Dumont #5SF forceps and remove the atria using Vannas spring surgical scissors.

***Positioning of the Heart:***

It is important to properly orient the heart under the microscope for successful exposure and removal of papillary muscles. The following steps describe positioning procedures:

1. Determine the anterior (frontal) and posterior (rear) views of the heart (Figure 1A), as these are key landmarks for proper positioning of the heart for performance of papillary muscle isolation procedure.

2. Before incision, the neonatal rat heart should be positioned with the right lateral part of the heart facing upward and towards the microscope objectives (Figure 1B).

3. Fix the position of the heart by immobilizing the apical portion of the heart with a curved Hartman Hemostat, with the outside of the curved hemostat jaws facing the bottom of the dish.

4. Place fine surgical forceps at the base of the heart to prevent it from slipping across the bottom of the dish during the isolation procedure. Lay a pair of hemostats (curved side up) perpendicular to the forceps for further stabilization.

5. Finally, rotate the dish such that the base of the heart is facing you, with the apical part facing in opposite direction (Figure 1B). This orientation will provide easy access to the right ventricle.

***Exposing of the Right Ventricular Papillary Muscles:***

An incision should be made from the basal part of the heart, taking most right approach from incision starting point towards apical part of the heart (Figure 1B).

1. In this procedure, elevate the surface of the heart (right lateral wall of right ventricle) with a Dumont #5SF forceps and insert scissors in the space formed in between ventricular walls, at the upper part of right ventricular chamber (incision starting point).

2. Once the tip of scissors is inside of right ventricular cavity, the incision should be continued from the initial incision starting point with a light, superficial cut through the entire thickness of the right ventricular wall towards apical part of the heart. In this procedure, the anterior ventricular wall should be pulled up with the forceps, and an incision line made along the right border, apical and left border of right ventricle, where the anterior wall meets the interventricular septum.

3. After completion of the 360° incision, the anterior wall of right ventricle becomes loose and can be removed. All three papillary muscles attached to the tricuspid valve leaflets will be located on the bottom of the exposed right ventricular chamber (on the surface of the intraventricular septum, from right ventricle view) (Figure 1C).

***Characterization of Right Ventricular Papillary Muscles:***

1. The anterior neonate rat papillary muscle is largest and characterized by a wider and shorter muscle body. It originates from the frontal part of the interventricular septum of right ventricular chamber and inserts chordae tendineae into anterior and posterior tricuspid valve leaflets.

2. The posterior papillary muscle is the second largest. It originates predominantly from the posterior part of interventricular septum of right ventricular chamber bordering the posterior wall, just ~1.5 mm aside from the anterior papillary muscle origination site. It is a thinner and more elongated muscle. Chordae tendineae insertion is fused into the septal and posterior leaflets of the tricuspid valve.

3. The third accessory neonate rat papillary muscle is the smallest, thinnest and shortest. When present, it arises from the septal region of the right ventricular chamber (between anterior and posterior papillary muscles) and inserts into anterior and septal tricuspid valve leaflets (Figure 1C). Due to its size the accessory papillary muscle is less suitable for contractile experiments than the anterior and posterior muscles; since it is difficult to attach to the force transducer, it will not be discussed further.

The total length of the anterior and posterior neonate rat papillary muscles (muscle body with chordae tendineae) is ~1.0 - 2.0 mm (varies from age of neonatal rat and muscle itself).

***Isolation of the Right Ventricular Papillary Muscles:***

Papillary muscles should be isolated without damage and stretching. Due to anatomical attachments of muscles to the right ventricular walls through the leaflets of the tricuspid valve, it is less traumatic to isolate anterior and posterior papillary muscles starting from the tricuspid valve site. Use the following steps to isolate the papillary muscles:

1. Gently pull the attachment site of tricuspid valve to the right ventricular walls with Dumont #5SF forceps, taking care not to stretch the papillary muscle.

2. Isolate the valve from surrounding tissue with fine surgical scissors, without damaging the chordae tendineae of the papillary muscle. Once the valve portion of the papillary muscle has been isolated from the surrounding connective tissue, the tendineae end of the papillary muscle becomes loose and can be detached.

3. In order to isolate the papillary muscle (wide muscular end) from the ventricular wall without damaging the muscle bundles, use scissors to penetrate the ventricular wall (intraventricular septum) close (1 mm away) to papillary muscle fusion point and parallel to the direction of papillary muscle. After initial penetration point, incision should be started and continued deep and further into ventricular wall for a couple more millimeters (~2-3 mm) in length and (~2 mm) in depth, depending on original size of papillary muscle.

4. After isolation of the muscular end of the papillary muscle from the ventricular wall, it is ready to be transported to the experimental test chamber for attachment to the force transducer and used for contractile experiments.
